# Bleeding After Central Venous Catheter Placement in a Patient With Undiagnosed Acquired Hemophilia A: A Case Report

**DOI:** 10.7759/cureus.27444

**Published:** 2022-07-29

**Authors:** Hikari Noguchi, Hiroyuki Seki, Joho Tokumine, Harumasa Nakazawa, Tomoko Yorozu

**Affiliations:** 1 Anesthesiology, Kyorin University School of Medicine, Tokyo, JPN

**Keywords:** mechanical complication, activated partial thromboplastin time, ultrasound-guided central venous catheterization, difficult hemostasis, acquired hemophilia a

## Abstract

Acquired hemophilia A is a rare condition caused by autoantibodies against endogenous coagulation factor VIII, which results in spontaneous bleeding. Workup of a patient with difficult hemostasis after removing and placing a central venous catheter led to the diagnosis of acquired hemophilia A.

A 64-year-old man was transferred with an intramuscular right thigh mass. Initial biopsy at an outside facility showed degenerated muscle and coagula and he was transferred for incisional biopsy and definitive treatment. The patient had difficult venous access, and a right internal jugular venous catheter was placed. The catheter insertion site showed slow continuous bleeding. Achieving adequate hemostasis after removing the catheter was difficult, and a hematoma formed after the placement of an infraclavicular axillary venous catheter under ultrasound guidance. Coagulation studies revealed a prolonged activated partial thromboplastin time at 96 seconds. The patient was then diagnosed with acquired hemophilia A by enzyme-linked immunosorbent assay using anti-factor VIII antibodies.

Even if ultrasound-guided central venous catheterization is performed carefully, bleeding may occur in some patients, suggesting the possibility of coagulopathy. Decision-making for performing central venous catheterization requires extensive knowledge of coagulopathies to understand the causes of bleeding complications.

## Introduction

Acquired hemophilia A is a rare but potentially life-threatening bleeding disorder caused by autoimmune dysfunction [1-3]. The incidence of acquired hemophilia A in adults is estimated to be about 14.7 cases per million per year [2]. Autoantibodies against endogenous coagulation factor VIII (FVIII) neutralize its hemostatic function, which leads to spontaneous bleeding [2,3]. The diagnosis of acquired hemophilia A should be suspected in patients with an isolated prolonged activated partial thromboplastin time (APTT). Diagnosis of acquired hemophilia A can be confirmed by Bethesda assay, Nijmegen assay, or enzyme-linked immunosorbent assay using anti-FVIII antibodies [2,3]. Treatment is decided depending on the titer of the FVIII inhibitor, and the site and severity of the bleeding. Activated prothrombin complex concentrate, recombinant activated FVII, and human or porcine FVIII are used as hemostatic agents [2,3]. Corticosteroids alone or in combination with immunosuppressants are considered to induce remission [2-4]. Infection is a major cause of death in patients with acquired hemophilia A after induction of remission [4].

It was difficult to achieve hemostasis after removing and placing a central venous catheter in a patient, which led to evaluation and the diagnosis of acquired hemophilia A.

## Case presentation

**Figure 1:**
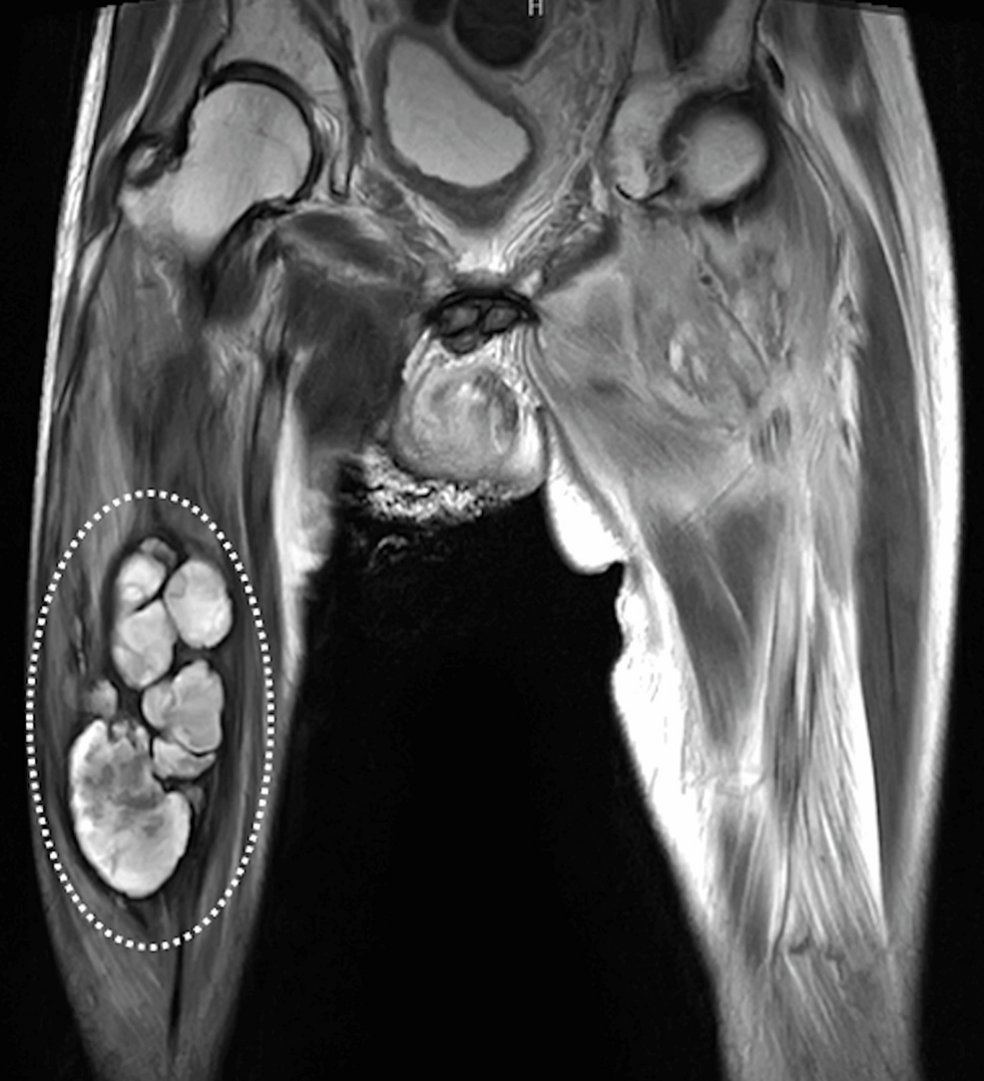
Intramuscular mass in the right thigh The dotted white oval shows an intramuscular mass in the right thigh on a T2-weighted magnetic resonance image.

A 64-year-old man presented to an outside facility with swelling of the right thigh. The patient had a history of urolithiasis. A computed tomography scan revealed a large intramuscular mass in the right thigh (Figure 1).

A biopsy showed degenerated muscle tissue and intramuscular hematoma. Laboratory data showed anemia without remarkable findings, and the patient was treated with red blood cell transfusions. With dry skin and blisters on the ankle joints, bullous pemphigoid was suspected. However, it was ruled out due to a failure to detect anti-BP180 autoantibody. Since the patient had difficult peripheral venous access, a right internal jugular venous catheter was placed. The catheter insertion site had continuous bloody drainage and the gauze dressing had to be replaced several times daily.

**Figure 2:**
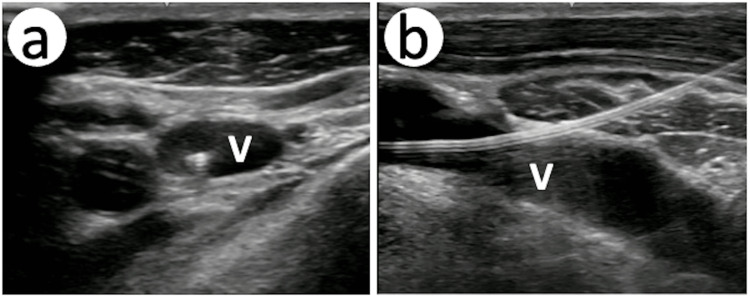
Infraclavicular axillary venous catheterization A: The guidewire is inserted in the infraclavicular axillary vein (short-axis view). The guidewire position is also verified in the long-axis view, the dilator is inserted and removed, and then the catheter is placed. B: The catheter is shown in the infraclavicular axillary vein without any hematoma. V: infraclavicular axillary vein.

The patient was transferred for further evaluation. On the day of admission, consultation was obtained for the anesthesiologist to replace the central venous catheter. The right internal jugular venous catheter was removed due to a suspected fistula. Despite 15 minutes of manual compression, the catheter site became swollen. The site was directly compressed for another 30 minutes, and no more swelling was observed. Another catheter was placed in the right infraclavicular axillary vein under ultrasound guidance without complications (Figure 2).

Although no bleeding was observed at the new catheter insertion site for two hours, the catheter was removed by a night duty physician due to obvious swelling at the catheter insertion site. Results of the coagulation studies revealed an extensively prolonged APTT (96 seconds), with prothrombin time and international normalized ratio (PT-INR) within normal limits (0.98). After further examinations by a hematologist, the patient was diagnosed with acquired hemophilia A and treated with steroids. The thigh mass and anemia resolved. Written informed consent was obtained from the patient for publication.

## Discussion

The diagnosis of acquired hemophilia A is usually delayed due to a lack of knowledge of this rare disease. Most patients with acquired hemophilia A have coagulation abnormalities, but some patients are asymptomatic with only prolonged APTT. In the presented patient, the only symptoms were an intramuscular mass and bloody exudate at the insertion site of an internal jugular vein catheter. Hematoma formation after removing and placing a central venous catheter made the anesthesiologist aware of a coagulation disorder.

In general, the differential diagnosis of isolated APTT prolongation was reported to be unfamiliar to anesthesiologists [5]. One reason for this may be the diversity of causes of mildly elevated APTT [6]. The causes of isolated prolonged APTT include lupus anticoagulant, anticoagulant therapy (mainly unfractionated heparin or argatroban), deficiency of coagulation factors (hemophilia), liver failure, excessive bleeding, disseminated intravascular coagulation, and fibrinogen deficiency [7,8]. Acquired hemophilia A has been known to be associated with other hematologic diseases, such as myelofibrosis, myelodysplastic syndrome, and autoimmune hemolytic anemia [9]. It is unclear whether this patient’s anemia was related to other hematologic diseases after receiving steroid therapy for acquired hemophilia A. Acquired hemophilia A has also been known to be secondary and/or comorbid with malignancies, which makes it difficult to establish a definitive diagnosis [9]. A simple diagnosis of acquired hemophilia A may reduce morbidity and mortality in patients with acquired hemophilia A. A diagnostic algorithm for unexplained bleeding with isolated prolongation of the APTT has been previously proposed [10].

Soon after ultrasound guidance was introduced as an adjunct to central venous catheter placement, it was thought to be useful to prevent complications of central venous catheter placement in patients with coagulation abnormalities [11]. Recently, ultrasound-guided central venous catheter placement has become the standard technique. A recent guideline imposes restrictions on coagulation studies as a requirement for ultrasound-guided central venous catheterization, which includes platelet count ≥ 5.0 × 104/μl, PT-INR ≤ 1.8, or APTT ≤ 1.3 times the upper normal limit [12]. However, there is no clear evidence to support these thresholds [13]. The prevalence of hematoma formation has been associated with insertion techniques [14]. The incidence of hematomas after central venous catheter placement was reported to be decreased after introducing the ultrasound-guided technique instead of the landmark technique [14].

In one systematic review [13], a platelet count below 5.0 × 104/μl and prolonged APTT were not associated with bleeding or hematoma formation. The impact of a prolonged PT-INR is controversial, with some reports suggesting that the PT-INR above 1.5 is associated with hematoma formation and others suggesting that it is not [13]. In the present patient, acquired hemophilia A, which is a coagulopathy with prolonged APTT, may result in bloody drainage or hematoma formation at the site of indwelling central venous catheters. Even though ultrasound-guided central venous catheterization is performed carefully, bleeding is not prevented in some patients with an underlying coagulopathy such as the present patient.

Patients who require a central venous catheter for venous access may have serious pathological conditions and generally present with coagulation abnormalities of varying severity. In situations of high mortality, immediate access to a central vein is justified, rather than waiting for further examinations for bleeding disorders. This case indicates that even careful insertion of central venous catheters under ultrasound guidance may cause hemorrhage. This case also raises a question regarding whether clinicians should screen for more rare coagulopathies before central venous catheter placement. If a safety margin of APTT is determined based on the evidence, the dilemma of whether or not to insert a central venous catheter in patients with coagulopathy may be resolved.

## Conclusions

This report provides valuable information to understand the safety margin of ultrasound-guided central venous catheter placement in a patient with coagulopathy, and highlights the clinical impact of previously undiagnosed acquired hemophilia A. More reports and extensive studies are needed to establish the safety margins for platelet count, international normalized ratio, and APTT values before ultrasound-guided central venous catheter placement. In conclusion, the decision-making process for the placement of a central venous catheter requires extensive knowledge of coagulopathies to understand bleeding complications.
